# Influenza vaccine coverage and factors associated with non-vaccination among caregiving and care-receiving adults in the Canadian Longitudinal Study on Aging (CLSA)

**DOI:** 10.1186/s12889-024-18372-6

**Published:** 2024-03-29

**Authors:** Katie Gravagna, Christina Wolfson, Nicole E. Basta

**Affiliations:** 1 Department of Epidemiology, Gillings School of Global Public Health, University of North Carolina at Chapel Hill, Chapel Hill, NC USA; 2 Department of Epidemiology, Biostatistics and Occupational Health, School of Population and Global Health, McGill University, Montreal, QC Canada; 3 Neuroepidemiology Research Unit, Research Institute of the McGill University Health Centre, Montreal General Hospital, Montreal, QC Canada

**Keywords:** Influenza, Vaccination, Canada, CLSA, Older adults

## Abstract

**Background:**

Influenza vaccination is recommended for those at increased risk of influenza complications and their household contacts to help reduce influenza exposure. Adults who require care often experience health issues that could increase the risk of severe influenza and have close contact with caregivers. Assessing influenza vaccination prevalence in caregivers and care recipients can provide important information about uptake.

**Objectives:**

We aimed to (1) estimate influenza non-vaccination prevalence and (2) assess factors associated with non-vaccination among caregivers aged ≥ 45 years and among care recipients aged ≥ 65 years.

**Methods:**

We conducted an analysis of cross-sectional data from the Canadian Longitudinal Study on Aging collected 2015–2018. We estimated non-vaccination prevalence and reported adjusted odds ratios with 95% confidence intervals from logistic regression models to identify factors associated with non-vaccination among caregivers and care recipients.

**Results:**

Of the 23,500 CLSA participants who reported providing care, 41.4% (95% CI: 40.8%, 42.0%) reported not receiving influenza vaccine in the previous 12 months. Among the 5,559 participants who reported receiving professional or non-professional care, 24.8% (95% CI: 23.7%, 26.0%) reported not receiving influenza vaccine during the same period. For both groups, the odds of non-vaccination were higher for those who had not visited a family doctor in the past year, were daily smokers, and those who identified as non-white.

**Discussion:**

Identifying groups at high risk of severe influenza and their close contacts can inform public health efforts to reduce the risk of influenza. Our results suggest sub-optimal influenza vaccination uptake among caregivers and care recipients. Efforts are needed to increase influenza vaccination and highlight the direct and indirect benefits for caregiver-care recipient pairs.

**Conclusion:**

The proportions of both caregivers and care recipients who had not been vaccinated for influenza was high, despite the benefits of vaccination. Influenza vaccination campaigns could target undervaccinated, high-risk groups to increase coverage.

**Supplementary Information:**

The online version contains supplementary material available at 10.1186/s12889-024-18372-6.

## Introduction

Seasonal influenza remains an important public health problem in Canada, where, on average, an estimated 12,200 hospitalizations and 3,500 deaths related to influenza occurred annually prior to the COVID-19 pandemic [1]. While the pandemic disrupted influenza transmission in both the 2020/2021 and 2021/2022 influenza seasons [2, 3], the 2021/2022 season had a proportion of visits to healthcare professionals due to influenza-like illness (ILI) similar to pre-pandemic levels [3], and during the 2022/2023 season, influenza had already resumed similar circulation to pre-pandemic years [4]. The prevention of influenza, and especially hospitalization and deaths due to influenza, remains a public health priority.

Vaccination against influenza reduces the risk of influenza infection, influenza transmission, and severe disease which could lead to hospitalization or death [5–7]. Vaccination benefits individuals at high risk of severe influenza directly by reducing their risk of influenza infection and associated negative outcomes such as hospitalization from ILI and both influenza-specific and all-cause mortality [8–13]. The National Advisory Committee on Immunization (NACI) “*particularly*” recommends annual influenza vaccination for groups at increased risk of influenza complications, including adults aged 65 years and older, to reduce the number of hospitalizations and deaths from influenza [14]. 

NACI also recommends influenza vaccination for household contacts of individuals at high risk of severe outcomes to prevent influenza illness and thus transmission by these contacts [15]. Close contacts of high risk individuals are important to consider in influenza prevention efforts given that close contacts may be a source of influenza transmission while at the same time close contacts of high-risk individuals have been found to influence seasonal influenza vaccination decision-making [16, 17]. Therefore, close contacts can impact both influenza risk and vaccination uptake for those at high risk of severe outcomes. Additionally, for care recipients who may be experiencing other comorbidities, recovery from influenza may be a lengthy process and may increase the care responsibilities for those who act as caregivers [18]. Despite this, vaccination coverage among caregivers and care recipients remains largely unexplored outside of studies that focus on healthcare workers or children [19–23]. 

The direct benefits of influenza vaccination in reducing an individual’s risk of infection and illness and the indirect benefits of vaccination in reducing the risk of transmission via reduced incidence is well recognized [24]. Vaccinating caregiver and care recipient pairs could directly reduce the risk of illness in both individuals and could have the added benefit of indirectly reducing the risk of influenza by reducing the risk of transmission during close contact.

Vaccination coverage in Canadian adults aged 65 years and older prior to the COVID-19 pandemic was estimated to be 65% for the 2015/2016 influenza season, 69% for the 2016/2017 season, and 70.7% for the 2017/2018 season, as reported by the Public Health Agency of Canada [1, 25, 26]. The degree to which those who serve as informal caregivers and their adult care-recipients receive influenza vaccine in a given season remains unknown in Canada. Prior studies of influenza vaccination uptake among those who provide or receive care have primarily focused on care in formal settings by health professionals such as in hospitals or care of children rather than adults [19–23]. 

To inform vaccination programs with the aim of increasing influenza vaccination coverage, it is necessary to obtain precise estimates of vaccination coverage and to determine the characteristics of those least likely to receive an influenza vaccination among subpopulations at high risk of severe outcomes and among those most likely to be the source of transmission to high-risk groups. While previous studies have reported characteristics that may be associated with influenza vaccination in Canadian adults in general [27–30], more insight is needed to determine uptake among specific groups. The Canadian Longitudinal Study on Aging (CLSA) provides a unique opportunity to assess uptake in caregivers and care recipients specifically as it is a large, national cohort study that includes information on influenza vaccination history along with a broad range of covariates that allow for the examination of associations with influenza non-vaccination in unique groups of interest across many different domains [31]. 

In this study, we aimed to (1) estimate the prevalence of influenza non-vaccination and assess factors associated with non-vaccination among caregivers aged 45 years and older and (2) estimate vaccination coverage and assess factors associated with non-vaccination among care recipients aged 65 years and older between 2015 and 2018.

## Materials and methods

### Data source

The CLSA recruited participants aged 45–85 years old from 2011 to 2015 using 3 sampling frames (the Canadian Community Health Survey, random-digit dialing, and provincial health registries) for the initial baseline visit. The CLSA is made up of 2 cohorts with many common elements. Participants in the Comprehensive cohort (*N* = 30,097 at baseline) attend in-person study visits and provide biological samples along with survey data. Participants in the Tracking cohort (*N* = 21,241 at baseline) provide survey data via computer-assisted telephone interviews only. Data collection among all cohort participants takes place every 3 years and is ongoing [32]. Recruitment was done across 7 provinces in the Comprehensive cohort (excluding Prince Edward Island, New Brunswick, and Saskatchewan) and across all 10 provinces in the Tracking cohort [32]. For the Comprehensive cohort, participants were randomly selected within age and sex strata from those who resided within 11 data collection site recruitment areas.

Individuals were deemed ineligible to participate in the CLSA for any of the following: residence on a federal First Nations reserve or other First Nations settlement; being a full-time member of the Canadian Armed Forces; residence in the 3 territories; residence in a long-term care institution; inability to respond in French or English; or cognitive impairment as determined by interviewers. Additional details regarding the CLSA study design and procedures has been published previously [31–33], and the CLSA surveys, protocols, and supporting documentation are publicly available on the CLSA website (clsa-elcv.ca).

We obtained approval to access de-identified CLSA data via the CLSA data access application process (Application Number: 2006029). Ethics approval for this analysis was obtained from the McGill University Institutional Research Board (Application Number: 21-02-048).

### Analytic Sample

This study is a cross-sectional analysis of the CLSA follow up 1 (FUP1) study visit that includes data collected from December 2015-July 2018. Participants in the Comprehensive (*N* = 27,765) and Tracking (*N* = 17,050) cohorts who had a yes or no response to the outcome variable (self-reported influenza vaccination status within the past 12 months) were included in these analyses. We identified survey questions that were asked in the same way to both Tracking and Comprehensive cohort participants. The two cohorts were combined for all analyses and only those variables collected from all participants were included in the analysis.

### Influenza vaccination status and covariates of interest

The variables included in these analyses were chosen *a priori* based on previously published studies that reported an association with influenza vaccination or their potential for identifying new target groups for vaccination [30, 34–36]. Data from the CLSA baseline assessment were used for race and education level whereas all other data were collected during the follow-up 1 visit. The CLSA variable coding used in these analyses can be found in Supplementary Table 1.

#### Influenza non-vaccination

Our outcome of interest “influenza non-vaccination” was determined by the self-reported response to the question “Have you had… Flu shot in the last 12 months”. Participants for whom the response was “Don’t Know/No Answer”, who “Refused”, or had a “Missing” response were excluded from the analysis due to the small proportion of respondents in these categories (0.003 of the Comprehensive cohort and 0.02 of the Tracking cohort). Self-reported influenza vaccination status has been validated for older adults in several populations, including the United States and Australia, and has been shown to have high sensitivity and moderate specificity compared to vaccination records [37, 38]. 

#### Sociodemographic variables and household characteristics

We investigated the association between 7 sociodemographic characteristics and influenza non-vaccination among caregivers and care recipients (Supplementary Table 1). Urban or rural categorization was dichotomized; participants classified as “Link to DA”, which indicates the cases where postal codes are linked at an insufficiently detailed level to provide urban/rural information, were considered rural [39]. For the province of residence, Ontario was selected as the reference category due to its historically higher vaccination coverage, including among those at high risk of severe outcomes, to aid in interpretation [40, 41]. 

Number of individuals (not including the participant) living in a household was determined using the variable “How many people, not including yourself, currently live in your household?”, where a response of 0 means the participant did not indicate anyone else currently lives in their household.

#### Chronic medical conditions

The type and number of chronic medical conditions (CMC) were also examined to evaluate the association between the presence of CMC and non-vaccination in those at high risk of severe outcomes (Supplementary Table 1). Participants were considered to have a CMC if they self-reported a physician diagnosis of any of the following categories of conditions which were selected based on NACI guidance about groups for whom influenza vaccination is particularly recommended [14]. For each CMC included in the CLSA dataset that aligned with the NACI guidelines, we categorized the condition into the following groups: heart disease, respiratory disorders, kidney disease or failure, asthma, diabetes, and cancer. Each was modeled as a separate binary variable (“yes”/“no”). Other CMC were combined into the variable “other CMC”. In addition, the variable “number of CMC” was derived by summing the number of CMC reported including the “other CMC” category, where applicable, for each participant.

#### Healthcare utilization, health perception, and health behaviors

Healthcare utilization factors (Supplementary Table 1) were also analyzed to assess their association with vaccination based on prior studies that identified the types of healthcare utilization that were associated with influenza vaccination in other settings [42, 43]. Healthcare visits provide an opportunity to engage with health providers who may make vaccination recommendations or offer vaccines.

Self-rated health was used in our analyses to assess the association between self-perceived health and influenza vaccination. Prior studies in Canada and the United States have found that health status, physical and psychological limitations, and other barriers to access like transportation associated with older age were also associated with influenza vaccination in groups at high risk of severe outcomes [11, 13, 40, 44, 45]. In our analyses, self-rated health was assessed by the response to the question “In general, would you say your health is excellent, very good, good, fair, or poor?”. Self-rated health has been validated [46, 47] as a measure of health in general health surveys.

To evaluate the impact of health-related behaviors that were previously identified as associated with influenza vaccination in older (≥ 65 year old) adult Canadians [40], participants were also categorized based on their self-reported smoking behavior, alcohol consumption, and exercise at the time of the survey (Supplementary Table 1).

#### Caregivers

Caregivers were defined as anyone aged 45 years and older who answered yes to the question, “During the past 12 months, have you provided any of the following types of assistance to another person because of a health condition or limitation?” for any of the following types of care (Supplementary Table 1): personal care, managing care, assistance with meals or housework, assistance with house maintenance or outdoor work, assistance with transportation, social/emotional assistance, mobility assistance, monetary assistance or financial management, other types of assistance. To evaluate influenza vaccination among individuals who act as caregivers, variables that help define the type of caregiving relationship (hours of care provided and if the care recipient lives with their caregiver) were also examined to determine if an association between different caregiver-care recipient relationships and influenza vaccine uptake could be identified.

#### Care recipients

We identified respondents aged 65 years and older who reported receiving at-home professional care, non-professional care, or both during the past 12 months by answering yes to receiving any of the following types of care listed in the question, “During the past 12 months, did you receive short-term or long-term assistance from family, friends, or neighbours because of a health condition or limitation that affects your daily life, for any of the following activities?” (Supplementary Table 1): personal care, medical care, managing care, assistance with activities, assistance with transportation, assistance with meal preparation, physical therapy, training and adaptation assistance, or other assistance. Participants who received both professional and non-professional care were included in both the non-professional and professional care groups for analysis.

**Table 1:** Demographic characteristics by influenza vaccination status among participants in the first follow-up visit (2015-18) of the Canadian Longitudinal Study on Aging: caregivers aged 45 years and older (*N* = 23,500).

	Received Influenza Vaccination in Last 12 Months; *N* = 13,771 (58.6%)	Did Not Receive Influenza Vaccination in Last 12 Months; *N* = 9,729 (41.4%)
	N(%)	N(%)
Sociodemographics		
Age		
46–54	1480(10.7)	2141(22.0)
55–64	4183(30.4)	4250(43.7)
65–74	4504(32.7)	2340(24.1)
75–84	3027(22.0)	869(8.9)
85–92	577(4.2)	129(1.3)
Province of Residence		
Newfoundland	707(5.1)	567(5.8)
Prince Edward Island	291(2.1)	153(1.6)
Nova Scotia	1389(10.1)	556(5.7)
New Brunswick	313(2.3)	183(1.9)
Quebec	1861(13.5)	2318(23.8)
Ontario	3255(23.6)	1944(20.0)
Manitoba	1233(9.0)	897(9.2)
Saskatchewan	346(2.5)	231(2.4)
Alberta	1590(11.5)	925(9.5)
British Columbia	2781(20.2)	1952(20.1)
Sex		
Male	6232(45.3)	4360(44.8)
Female	7534(54.7)	5363(55.1)
Urban or Rural		
Urban	11943(86.7)	8140(83.7)
Rural	1272(9.2)	1113(11.4)
Household Income		
< 20,000	482(3.5)	447(4.6)
≥20,000 to < 50,000	2902(21.1)	2077(21.3)
≥50,000 to < 100,000	4987(36.2)	3213(33.0)
≥100,000 to < 150,000	2424(17.6)	1808(18.6)
≥150,000	2122(15.4)	1653(17.0)
Education		
Less than secondary school graduation	669(4.9)	445(4.6)
Secondary school graduation, no post-secondary education	1310(9.5)	1025(10.5)
Some post-secondary education	1001(7.3)	742(7.6)
Post-secondary degree/diploma	10762(78.1)	7497(77.1)
Race		
White	13256(96.3)	9215(94.7)
Non-white	505(3.7)	501(5.1)

### Statistical analysis

To estimate influenza vaccination coverage among (1) caregivers aged 45 years and older and (2) care recipients aged 65 years and older, we calculated influenza non-vaccination prevalence in the previous 12 months for caregivers and care recipients by the presence of chronic medical conditions, healthcare utilization history, self-rated health, health behaviors, and sociodemographic factors.

The association between the independent variables and influenza non-vaccination was estimated using multivariable logistic regression. The adjusted odds ratios (aOR) along with 95% confidence intervals (95% CIs) were reported for the association between each covariate included in each model and not having received influenza vaccine in the past 12 months.

To assess factors associated with non-vaccination for the care recipient and caregiver groups, we created 3 models: a basic model including demographic factors and self-reported chronic medical conditions; a larger model including those variables plus total CMC and other healthcare- and care-related variables; and a full model including all covariates of interest (Supplementary Tables 2–3). The results of the fully adjusted model are reported in the results section.

For chronic medical conditions by type, type of healthcare utilization in the past 12 months, and care or assistance received, the reference category is those without the characteristic or condition of interest (for example, the reference category for “family doctor contact” is those who did not have family doctor contact in the past 12 months. For all other variables, the reference categories are identified in Tables 3 and 4.

Due to the complex sampling techniques used by the CLSA, inflation and analytic weights have been made available for the baseline datasets. However, baseline weights are not applicable for cross-sectional analyses of the FUP1 data and sampling weights for FUP1 were not available at the time of the analyses thus sampling weights were not used here. We stratified by age and included the variables of sex and province of residence in our analyses as recommended by the CLSA [48]. 

We conducted a sensitivity analysis to determine if the prevalence of vaccination was significantly different between individuals who were asked about their annual influenza vaccination during each of the Canadian influenza seasons (November-April in each year) compared to those who were surveyed outside of each influenza season (May-October in each year) [1] using two-sample tests of proportions comparing these two groups across all survey years.

These analyses were conducted in R (version 1.3.1073) using the survey package.

## Results

### Participant demographics

In total, 44,372 participants were eligible for this analysis. Of the 44,372 participants in this study sample with a valid response to the outcome variable, 23,500 were classified as caregivers. The distribution of demographic characteristics for the 23,500 (53%) caregivers aged 45 years and older and the 5,559 (13%) care recipients aged 65 years and older by vaccination status are reported (Tables 1 and 2).

**Table 2:** Demographic characteristics by influenza vaccination status among participants in the first follow-up visit (2015-18) of the Canadian Longitudinal Study on Aging: care recipients aged 65 years and older (*N* = 5,559).

	Received Influenza Vaccination in Last 12 Months; *N* = 4,178 (75.2%)	Did Not Receive Influenza Vaccination in Last 12 Months; *N* = 1,381 (24.8%)
	N(%)	N(%)
Sociodemographics		
Age		
65–74	1617(38.7)	713(51.6)
75–84	1922(46.0)	503(36.4)
85–92	639(15.3)	165(11.9)
Province of Residence		
Newfoundland	178(4.3)	84(6.1)
Prince Edward Island	101(2.4)	23(1.7)
Nova Scotia	393(9.4)	72(5.2)
New Brunswick	102(2.4)	38(2.8)
Quebec	688(16.5)	346(25.1)
Ontario	937(22.4)	227(16.4)
Manitoba	368(8.8)	132(9.6)
Saskatchewan	97(2.3)	38(2.8)
Alberta	497(11.9)	125(9.1)
British Columbia	817(19.6)	295(21.4)
Sex		
Male	1716(41.1)	528(38.2)
Female	2458(58.8)	852(61.7)
Urban or Rural		
Urban	3651(87.4)	1167(84.5)
Rural	354(8.5)	143(10.4)
Household Income		
< 20,000	307(7.3)	180(13.0)
≥20,000 to < 50,000	1360(32.6)	545(39.5)
≥50,000 to < 100,000	1381(33.1)	369(26.7)
≥100,000 to < 150,000	429(10.3)	85(6.2)
≥150,000	228(5.5)	47(3.4)
Education		
Less than secondary school graduation	390(9.3)	160(11.6)
Secondary school graduation, no post-secondary education	483(11.6)	169(12.2)
Some post-secondary education	350(8.4)	131(9.5)
Post-secondary degree/diploma	2942(70.4)	912(66.0)
Race		
White	4063(97.2)	1316(95.3)
Non-white	113(2.7)	64(4.6)

### Influenza vaccination in caregivers aged 45 years and older

The prevalence of influenza non-vaccination in the past 12 months was 0.41 (95% CI: 0.41, 0.42) in this group. The proportion of participants who reported not being vaccinated decreased as age increased (Table 3, first column). Residents of Quebec had the highest prevalence of non-vaccination (0.55, 95% CI: 0.54, 0.57). Those with two or more additional members of their household had a higher prevalence of non-vaccination (0.51, 95% CI: 0.49, 0.52) than those with one or no additional members.

**Table 3:** Proportion unvaccinated and factors associated with non-vaccination status against seasonal influenza among participants in the first follow-up visit (2015-18) of the Canadian Longitudinal Study on Aging: caregivers aged 45 years and older (*N* = 23,500).

		Full Model *N* = 19,377
	Proportion Unvaccinated (95% CI)	aOR (95% CI)
Age (Years)		
85–92	0.18 (0.15, 0.21)	Ref
75–84	0.22 (0.21, 0.24)	1.42 (1.11, 1.82)
65–74	0.34 (0.33, 0.35)	2.43 (1.91, 3.09)
55–64	0.50 (0.49, 0.51)	4.71 (3.70, 6.00)
46–54	0.59 (0.58, 0.61)	6.29 (4.89, 8.11)
Province of Residence		
Ontario	0.37 (0.36, 0.39)	Ref
Newfoundland	0.45 (0.42, 0.47)	1.41 (1.22, 1.63)
Prince Edward Island	0.34 (0.30, 0.39)	0.75 (0.59, 0.97)
Nova Scotia	0.29 (0.27, 0.31)	0.58 (0.51, 0.67)
New Brunswick	0.37 (0.33, 0.41)	0.83 (0.66, 1.05)
Quebec	0.55 (0.54, 0.57)	1.94 (1.76, 2.14)
Manitoba	0.42 (0.40, 0.44)	1.09 (0.97, 1.24)
Saskatchewan	0.40 (0.36, 0.44)	1.00 (0.81, 1.23)
Alberta	0.37 (0.35, 0.39)	0.95 (0.85, 1.07)
British Columbia	0.41 (0.4, 0.43)	1.21 (1.10, 1.33)
Sex		
Male	0.41 (0.40, 0.42)	Ref
Female	0.42 (0.41, 0.42)	1.14 (1.07, 1.22)
Urban or Rural		
Rural	0.47 (0.45, 0.48)	Ref
Urban	0.41 (0.40, 0.41)	0.76 (0.70, 0.83)
Household Income (Canadian Dollars)		
<20,000	0.48 (0.45, 0.51)	Ref
≥20,000 to < 50,000	0.42 (0.40, 0.43)	0.87 (0.73, 1.04)
≥50,000 to < 100,000	0.39 (0.38, 0.40)	0.63 (0.53, 0.75)
≥100,000 to < 150,000	0.43 (0.41, 0.44)	0.57 (0.47, 0.69)
≥150,000	0.44 (0.42, 0.45)	0.50 (0.41, 0.60)
Education		
Less than secondary school graduation	0.40 (0.37, 0.43)	Ref
Secondary school graduation, no post-secondary education	0.44 (0.42, 0.46)	1.09 (0.91, 1.31)
Some post-secondary education	0.43 (0.40, 0.45)	1.11 (0.92, 1.34)
Post-secondary degree/diploma	0.41 (0.40, 0.42)	0.93 (0.79, 1.09)
Race		
White	0.41 (0.40, 0.42)	Ref
Non-white	0.50 (0.47, 0.53)	1.42 (1.22, 1.65)
CMC by Type		
Heart Disease	0.29 (0.27, 0.30)	0.90 (0.79, 1.02)
Respiratory disorders	0.31 (0.29, 0.34)	0.85 (0.73, 0.99)
Kidney Disease or Failure	0.33 (0.30, 0.37)	0.95 (0.78, 1.15)
Asthma	0.34 (0.33, 0.36)	0.73 (0.65, 0.83)
Diabetes	0.33 (0.31, 0.34)	0.79 (0.70, 0.89)
Cancer	0.31 (0.29, 0.32)	0.89 (0.79, 1.00)
Other CMC	0.34 (0.33, 0.35)	0.88 (0.78, 0.99)
Number in Household Besides Participant		
0	0.39 (0.37, 0.40)	Ref
1	0.38 (0.38, 0.39)	1.01 (0.93, 1.11)
≥ 2	0.51 (0.49, 0.52)	1.21 (1.09, 1.35)
Hours of Care Provided Weekly		
1–20	0.42 (0.41, 0.43)	Ref
21–40	0.39 (0.37, 0.41)	0.93 (0.82, 1.05)
41+	0.39 (0.36, 0.41)	1.01 (0.88, 1.15)
Location of Care Recipients		
Living in Your Household	0.36 (0.35, 0.37)	Ref
Living in Another Household	0.43 (0.43, 0.44)	1.16 (1.07, 1.26)
Living in a Health Care Institution	0.41 (0.39, 0.43)	1.13 (1.00, 1.27)
Number of CMC		
0	0.51 (0.50, 0.53)	Ref
1	0.42 (0.41, 0.43)	0.97 (0.86, 1.09)
≥ 2	0.30 (0.29, 0.31)	0.83 (0.66, 1.03)
Type of Healthcare Utilization, Past 12 months		
Family Doctor Contact	0.39 (0.38, 0.40)	0.53 (0.47, 0.59)
Specialist Contact	0.36 (0.35, 0.37)	0.81 (0.76, 0.86)
Self-Rated Health		
Excellent	0.45 (0.43, 0.46)	Ref
Very Good	0.42 (0.41, 0.43)	0.99 (0.91, 1.08)
Good	0.41 (0.40, 0.42)	0.96 (0.87, 1.06)
Fair	0.36 (0.34, 0.38)	0.88 (0.76, 1.01)
Poor	0.32 (0.28, 0.37)	0.78 (0.59, 1.03)
Exercise Past Week		
None or Seldom	0.41 (0.40, 0.41)	Ref
Sometimes or Often	0.44 (0.42, 0.45)	1.00 (0.93, 1.08)
Smoking Currently		
Not at All	0.40 (0.40, 0.41)	Ref
Occasionally	0.52 (0.47, 0.57)	1.01 (0.79, 1.29)
Daily	0.56 (0.53, 0.58)	1.44 (1.25, 1.65)
Alcohol Past 12 Months		
Never	0.41 (0.39, 0.43)	Ref
Occasionally	0.42 (0.41, 0.44)	1.07 (0.94, 1.22)
Regular	0.41 (0.41, 0.42)	0.92 (0.84, 1.02)

Table 3 also presents the results of the logistic regression analyses for caregivers aged 45 years and older. Younger age was associated with higher odds of being unvaccinated at the time of the survey, particularly for those aged 64 years and younger after controlling for all other variables (Table 2). Those who identified as non-white had higher odds of being unvaccinated (1.42, 95% CI: 1.22, 1.65) than those who identified as white. Daily smokers had higher odds of non-vaccination (1.44, 95% CI: 1.25, 1.65) than occasional or never smokers. In contrast, individuals who reported contact with a family doctor or who reported contact with a specialist in the past 12 months had lower odds of being unvaccinated (0.53, 95% CI: 0.47, 0.59 and 0.81, 95% CI: 0.76, 0.86, respectively) than those without medical contact. Higher household income was associated with lower odds of non-vaccination. Those living in urban areas had lower odds of non-vaccination (0.76, 95% CI: 0.70, 0.83) than those in rural areas.

### Influenza vaccination in care recipients aged 65 years and older

Among care recipients (*N* = 5,559), the prevalence of non-vaccination over the past 12 months was 0.25 (95% CI: 0.24, 0.26). Vaccination coverage prevalence was similar for all types of chronic medical conditions, and vaccine coverage did not differ by self-rated health (Table 4, first column). Vaccine coverage varied by household income: among those with the lowest income (< 20,000 Canadian dollars) the non-vaccination prevalence was 0.37 (95% CI: 0.33, 0.41), while the prevalence was 0.17 (95% CI: 0.13, 0.22) for the highest-income group of ≥ 150,000 Canadian dollars.

**Table 4:** Proportion unvaccinated and factors associated with non-vaccination status against seasonal influenza among participants in the first follow-up visit (2015-18) of the Canadian Longitudinal Study on Aging: care recipients aged 65 years and older (*N* = 5,559).

		Full Model *N* = 4,521
	Proportion Unvaccinated (95% CI)	aOR (95% CI)
Age (Years)		
85–92	0.21 (0.18, 0.23)	Ref
75–84	0.21 (0.19, 0.22)	1.05 (0.83, 1.34)
65–74	0.31 (0.29, 0.32)	1.88 (1.48, 2.39)
Province of Residence		
Ontario	0.20 (0.17, 0.22)	Ref
Newfoundland	0.32 (0.26, 0.38)	1.99 (1.41, 2.82)
Prince Edward Island	0.19 (0.12, 0.25)	0.84 (0.49, 1.43)
Nova Scotia	0.15 (0.12, 0.19)	0.71 (0.50, 1.01)
New Brunswick	0.27 (0.20, 0.35)	1.13 (0.70, 1.81)
Quebec	0.33 (0.31, 0.36)	1.90 (1.52, 2.38)
Manitoba	0.26 (0.23, 0.30)	1.16 (0.86, 1.58)
Saskatchewan	0.28 (0.21, 0.36)	1.45 (0.90, 2.33)
Alberta	0.20 (0.17, 0.23)	0.98 (0.74, 1.30)
British Columbia	0.27 (0.24, 0.29)	1.45 (1.16, 1.81)
Sex		
Male	0.24 (0.22, 0.25)	Ref
Female	0.26 (0.24, 0.27)	1.09 (0.93, 1.27)
Urban or Rural		
Rural	0.29 (0.26, 0.32)	Ref
Urban	0.24 (0.23, 0.25)	0.77 (0.62, 0.94)
Household Income (Canadian Dollars)		
< 20,000	0.37 (0.33, 0.41)	Ref
≥20,000 to < 50,000	0.29 (0.27, 0.31)	0.78 (0.61, 0.99)
≥50,000 to < 100,000	0.21 (0.19, 0.23)	0.52 (0.40, 0.68)
≥100,000 to < 150,000	0.17 (0.13, 0.20)	0.38 (0.27, 0.55)
≥150,000	0.17 (0.13, 0.22)	0.42 (0.27, 0.64)
Education		
Less than secondary school graduation	0.29 (0.25, 0.33)	Ref
Secondary school graduation, no post-secondary education	0.26 (0.23, 0.29)	0.93 (0.68, 1.26)
Some post-secondary education	0.27 (0.23, 0.31)	1.28 (0.92, 1.78)
Post-secondary degree/diploma	0.24 (0.22, 0.25)	0.94 (0.73, 1.22)
Race		
White	0.24 (0.23, 0.26)	Ref
Non-white	0.36 (0.29, 0.43)	2.04 (1.39, 2.99)
CMC by Type		
Heart Disease	0.22 (0.20, 0.25)	0.87 (0.72, 1.07)
Respiratory Disorders	0.20 (0.17, 0.23)	0.63 (0.49, 0.81)
Kidney Disease or Failure	0.21 (0.17, 0.25)	0.78 (0.57, 1.05)
Asthma	0.23 (0.20, 0.26)	0.97 (0.77, 1.22)
Diabetes	0.23 (0.21, 0.25)	0.87 (0.72, 1.06)
Cancer	0.22 (0.20, 0.24)	0.88 (0.72, 1.06)
Other CMC	0.24 (0.22, 0.25)	0.88 (0.71, 1.09)
Number of CMC		
0	0.29 (0.26, 0.32)	Ref
1	0.28 (0.25, 0.30)	1.10 (0.85, 1.44)
≥ 2	0.22 (0.21, 0.24)	0.92 (0.62, 1.36)
Care or Assistance Received		
Professional	0.23 (0.21, 0.24)	0.89 (0.74, 1.06)
Non-Professional	0.25 (0.24, 0.27)	1.08 (0.87, 1.34)
Type of Healthcare Utilization, Past 12 Months		
Family Doctor Contact	0.24 (0.23, 0.25)	0.61 (0.41, 0.90)
Specialist Contact	0.23 (0.22, 0.24)	0.71 (0.59, 0.86)
Hospitalization History	0.25 (0.23, 0.27)	1.16 (0.98, 1.36)
Self-Rated Health		
Excellent	0.28 (0.24, 0.32)	Ref
Very Good	0.25 (0.23, 0.27)	0.99 (0.75, 1.29)
Good	0.24 (0.22, 0.26)	0.94 (0.72, 1.23)
Fair	0.25 (0.23, 0.28)	1.10 (0.81, 1.48)
Poor	0.21 (0.17, 0.26)	0.84 (0.56, 1.25)
Number in Household Besides Participant		
0	0.27 (0.25, 0.29)	Ref
1	0.23 (0.21, 0.25)	0.99 (0.83, 1.17)
≥ 2	0.26 (0.22, 0.30)	1.11 (0.85, 1.45)
Exercise Past Week		
None or Seldom	0.24 (0.23, 0.26)	Ref
Sometimes or Often	0.27 (0.24, 0.31)	1.08 (0.88, 1.34)
Smoking Currently		
Not at All	0.24 (0.23, 0.25)	Ref
Occasionally	0.33 (0.19, 0.48)	1.43 (0.66, 3.12)
Daily	0.34 (0.29, 0.40)	1.46 (1.06, 1.99)
Alcohol Past 12 Months		
Never	0.27 (0.25, 0.30)	Ref
Occasionally	0.28 (0.25, 0.31)	1.07 (0.85, 1.35)
Regular	0.23 (0.22, 0.25)	0.90 (0.74, 1.09)

Table 4 presents the results of the logistic regression analyses for care recipients aged 65 years and older. The odds of influenza non-vaccination in the past 12 months were higher in those aged 65–74 compared to those aged 85–94 after controlling for all other variables (1.88, 95% CI: 1.48, 2.39) Those who identified as non-white had higher odds of being unvaccinated (2.04, 95% CI: 1.39, 2.99) than those who identified as white. Compared to occasional or never smokers, daily smokers had higher odds of non-vaccination (1.46, 95% CI: 1.06, 1.99). Increasing income was generally associated with lower odds of non-vaccination. The odds of non-vaccination were lower in those who reported contact with a family doctor (0.61, 95% CI: 0.41, 0.90) or specialist (0.71, 95% CI: 0.59, 0.86) in the past 12 months compared to those without contact.

### Sensitivity analysis

For caregivers (*N* = 23,500), the proportions of influenza non-vaccination by cohort were not significantly different between individuals surveyed during the typical Canadian influenza season from November-April (proportion = 0.51) and individuals surveyed outside those months (proportion = 0.49) (Comprehensive Cohort, *p* = 0.17; Tracking Cohort, *p* = 0.33). For care recipients (*N* = 5,559), the proportions of influenza non-vaccination by cohort were also not significantly different between individuals surveyed during the typical Canadian influenza season (proportion = 0.50) and individuals surveyed outside those months (proportion = 0.50) (Comprehensive Cohort, *p* = 0.93; Tracking Cohort, *p* = 0.12).

## Discussion

Influenza vaccination remains a critical tool for reducing preventable morbidity and mortality due to influenza. Vaccination recommendations often target groups at risk of severe outcomes, as well as their household contacts due to the risk of influenza transmission. Our study found that about 2 out of 5 caregivers aged 45 and older did not receive influenza vaccine during the survey period 2015–2018, and nearly 1 out of 4 care recipients aged 65 years and older did not receive influenza vaccine during this period either. Younger individuals in both groups were less likely to be vaccinated; for example, among caregivers aged 45–64 years old, over half reported being unvaccinated. We found that having visited a family doctor in the 12 months prior to the survey was associated with higher vaccination for both groups.

Caregivers often provide essential and critical care for individuals who need them. In doing so, caregivers may have close, frequent, and sustained contact with those at high risk of severe outcomes. We considered this group as a strong potential target for influenza vaccination campaigns, where efforts to increase vaccination uptake could directly benefit caregivers by reducing their risk of influenza and indirectly benefit those they care for by reducing the risk their caregiver may be unavailable due to illness plus reducing the risk of transmission from a close contact. The prevalence of vaccination among caregivers was particularly low. Our findings on vaccination in caregivers highlight the importance of ensuring that caregivers are aware of the multiple direct and indirect benefits of influenza vaccination. Influenza transmission between caregivers and care recipients could be a significant issue for both groups: caregivers may be unable to provide the same level of care if they become ill, while care recipients may experience a higher prevalence of morbidity and mortality if they become ill. It is also important for caregivers to get vaccinated as part of efforts to attend to their own health and well-being. Influenza vaccination of both caregivers and care recipients provides the dual benefit of reducing the risk to both during their close contact.

A study that analyzed cross-sectional data from the 2009 Behavioral Risk Factor Surveillance System in the United States found that those with functional limitations were more likely to have received an influenza vaccination in the past 12 months and have a regular healthcare provider [49]. This may indicate a higher likelihood to seek care due to these limitations, leading to greater exposure to vaccination recommendations from providers.

One notable finding of this study is that caregivers and care recipients who have had contact with a family doctor during the past 12 months had lower odds of being unvaccinated after controlling for all other variables. Having a family doctor was also shown to be associated with influenza vaccination in a 2009 study of 134,072 Canadians with chronic respiratory disease [43]. The impact of healthcare provider recommendations on influenza vaccination has been well documented. Ensuring that individuals have access to healthcare provider visits when needed may help increase vaccination coverage further.

For care recipients, the number of chronic medical conditions (CMC) and lower self-rated health was not associated with non-vaccination, and the trend between declining health status and vaccination was moderate for caregivers as well. This is in contrast to several previous studies in older adult Canadians that found that self-rated health and presence of CMC were associated with influenza vaccination [12, 28, 30, 40, 42]. Of these five studies, only two looked at any form of physician visit (Campitelli *et al.* included outpatient clinic visits in the past 3 years and Roy *et al.* included having or not having had a family doctor in their analyses respectively). None of the studies evaluated associations with specialist health care visits. Our results suggest that health status may not be associated with vaccination once more in-depth evaluations of specialist and family doctor contact are included in the analysis. Rather, after accounting for these factors, our results suggest that those who have had contact with a family doctor or specialist in the past 12 months have lower odds of being unvaccinated and those who smoke daily have higher odds of being unvaccinated.

Our study contributes new insight into vaccination uptake among caregivers and care recipients and factors associated with a lack of vaccination which can be used to identify opportunities for public health programs to close the existing gaps in influenza vaccination coverage and prevent transmission within these groups. Care recipients without contact with a family doctor and caregivers who are household contacts of those at high risk of severe outcomes may be unaware of their recommendation to be vaccinated against influenza and the accompanying benefits: public health outreach efforts could be aimed at these important groups for influenza prevention.

Our analysis has several unique strengths given the large sample of tens of thousands of older Canadians included in the CLSA, the wide range of detailed socio-demographic and health data collected, and the ability to analyze the association between multiple factors and vaccination status simultaneously. Consequently, the results go beyond and complement routinely collected vaccination survey data and provide additional insight. However, our study also has limitations that could be improved upon in future studies. For example, many variables, including the vaccination status, are based on self-report, although the outcome of self-reported influenza vaccination has been validated in prior studies and the time period (previous 12 months) was relatively short for recall [37, 38]. While the CLSA sampling was designed to balance generalizability to the Canadian population and feasibility, the approach does not include all groups, most notably those residing on a First Nations reserve and those institutionalized individuals, both of whom were not eligible during recruitment [50]. Follow-up studies should investigate influenza vaccine uptake in these groups as our results may not be generalizable. Applicable survey weights were not available for a cross-sectional analysis of FUP1; while we stratified by age and included the variables of sex and province of residence in our analyses as recommended by the CLSA [48], these study findings may not be generalizable to the target population. We also do not have clear information on the professional status of someone who identifies as a caregiver (e.g., if they are a professional paid for their caregiving services). No information was collected to identify reasons for non-vaccination thus limiting our ability to infer which factors shaped the vaccination rates observed; further investigation into the mechanisms behind non-vaccination is certainly necessary. At the time of this analysis, no information was available about influenza vaccination over time or during other influenza seasons, either. Changes in vaccination status over time could better elucidate trends among caregivers and care recipients.

## Conclusion

Given that our analyses indicate that an estimated 2 out of 5 adult caregivers and 1 out of 4 older adult care-recipients were not vaccinated against influenza in the year prior to the survey, efforts are needed to increase uptake in these groups. For both groups, encouraging influenza vaccination in younger age groups may be particularly beneficial in increasing coverage, as is specifically engaging with care recipients and caregivers who rarely attend health care provider visits to ensure that they are aware of vaccination recommendations and benefits of vaccination. Caregivers and care-recipients are two important groups to consider when planning efforts to reduce the risk of influenza and reduce the morbidity and mortality associated with severe disease.

### Electronic supplementary material

Below is the link to the electronic supplementary material.

Supplementary Material 1

## Data Availability

Data are available from the Canadian Longitudinal Study on Aging (www.clsa-elcv.ca) for researchers who meet the criteria for access to de-identified CLSA data.
