# Supplementary material for: Influenza vaccine coverage and factors associated with non-vaccination among caregiving and care-receiving adults in the Canadian Longitudinal Study on Aging (CLSA)
Source: BMC Public Health. 2024 Mar 29;24:924. doi: 10.1186/s12889-024-18372-6 (PMC10981287; doi:10.1186/s12889-024-18372-6)
Supplement: Supplementary file 1 — Supplementary Material 1 [file 12889_2024_18372_MOESM1_ESM.docx]

**S1 Table. CLSA questionnaire descriptions, variables, responses options, and variable categorization**

| **CLSA Survey Question and Variable Label** | **CLSA Code and Response Options 🡪 Variable Categorization for Analysis** |
| --- | --- |
| **Flu Shot**  Have you had… Flu shot in the last 12 months  PHB_FLUV_COF1^a^  PHB_FLUV_TRF1^a^ | 1 = Yes  2 = No  9 = Refused 🡪 NA  -88888 = Missing 🡪 NA |
| **Participant age**  Age at FU1 (in years)  AGE_NMBR_COF1  AGE_NMBR_TRF1 | 46-54, 55-64, 65-74, 75-84, 85-92 |
| **Province of residence**  WGHTS_PROV_COF1  WGHTS_PROV_TRF1 | Newfoundland, Prince Edward Island, Nova Scotia, New Brunswick,  Quebec, Ontario, Manitoba,  Saskatchewan, Alberta, British Columbia |
| **Sex**  What was your sex at birth?  SDC_BTHSEX_COF1  SDC_BTHSEX_TRF1 | 1 = Male  2 = Female  8 = Don't know/No answer 🡪 NA  9 = Refused 🡪 NA  -88888 = Missing 🡪 NA |
| **Urban/ rural**  SDC_URBAN_RURAL_COF1  SDC_URBAN_RURAL_TRF1 | 0 = Rural area 🡪 Rural  1 = Urban core 🡪 Urban  2 = Urban fringe 🡪 Urban  3 = Rural fringe in CMA/CAs 🡪 Rural  4 = Urban Areas out CMA/CAs 🡪 Urban  5 = Rural fringe out CMA/CAs 🡪 Rural  6 = Secondary urban core 🡪 Urban  9 = Link to DA 🡪 Rural  -88888 = Missing 🡪 NA |
| **Household Income**  What is your best estimate of the total household income received by all household members, from all sources, before taxes and deductions, in the past 12 months?  INC_TOT_COF1  INC_TOT_TRF1 | 1 = Less than $20,000  2 = $20,000 or more, but less than $50,000  3 = $50,000 or more, but less than $100,000  4 = $100,000 or more, but less than $150,000  5 = $150,000 or more  8 = Don't know/No answer 🡪 NA  9 = Refused 🡪 NA  -88888 = Missing 🡪 NA |
| **Education level**  Highest Level of Education - Respondent, 4 Levels  ED_UDR04_COM  ED_UDR04_TRM | 1 = Less than secondary school graduation  2 = Secondary school graduation, no post-secondary education  3 = Some post-secondary education  4 = Post-secondary degree/diploma  9 = At least one required question was not answered 🡪 NA |
| **Cultural / Racial Background**  SDC_DCGT_COM  SDC_DCGT_TRM | 1 = white only 🡪 white  2 = Black only 🡪 Non-white  3 = Korean only 🡪 Non-white  4 = Filipino only 🡪 Non-white  5 = Japanese only 🡪 Non-white  6 = Chinese only 🡪 Non-white  7 = South Asian only 🡪 Non-white  8 = Southeast Asian only 🡪 Non-white  9 = Arab only 🡪 Non-white  10 = West Asian only 🡪 Non-white  11 = Latin American only 🡪 Non-white  12 = Other racial or cultural origin (only) 🡪 Non-white  13 = Multiple racial or cultural origins 🡪 Non-white  99 = Required question was not answered 🡪 NA |
| **Type of CMC - Heart Disease**  Has a doctor ever told you that you have heart disease (including congestive heart failure or CHF)?  CCC_HEART_COF1  CCT_HEART_TRF1 | 1 = Yes  2 = No  8 = Don't know/No answer 🡪 NA  9 = Refused 🡪 NA  -88880 = Did not complete a DCS visit 🡪 NA  -88888 = Missing 🡪 NA |
| **Type of CMC – Respiratory Disorders**  Has a doctor told you that you have/had any of the following: emphysema, chronic bronchitis, chronic obstructive pulmonary disease (COPD), or chronic changes in lungs due to smoking?  CCC_COPD_COF1  CCT_COPD_TRF1 | 1 = Yes  2 = No  8 = Don't know/No answer 🡪 NA  9 = Refused 🡪 NA  -88880 = Did not complete a DCS visit 🡪 NA  -88888 = Missing 🡪 NA |
| **Type of CMC - Kidney Disease or Failure**  Has a doctor ever told you that you have kidney disease or kidney failure?  CCC_KIDN_COF1  CCT_KIDN_TRF1 | 1 = Yes  2 = No  8 = Don't know/No answer 🡪 NA  9 = Refused 🡪 NA  -88880 = Did not complete a DCS visit 🡪 NA  -88888 = Missing 🡪 NA |
| **Type of CMC - Asthma**  Has a doctor ever told you that you have asthma?  CCC_ASTHM_COF1  CCT_ASTHM_TRF1 | 1 = Yes  2 = No  8 = Don't know/No answer 🡪 NA  9 = Refused 🡪 NA  -88880 = Did not complete a DCS visit 🡪 NA  -88888 = Missing 🡪 NA |
| **Type of CMC - Diabetes**  Has a doctor ever told you that you have diabetes, borderline diabetes or that your blood sugar is high?  DIA_DIAB_COF1  CCT_DIAB_TRF1 | 1 = Yes  2 = No  8 = Don't know/No answer 🡪 NA  9 = Refused 🡪 NA  -88888 = Missing 🡪 NA  -88880 = Did not complete a DCS visit 🡪 NA |
| **Type of CMC - Cancer**  Has a doctor ever told you that you had cancer?  CCC_CANC_COF1  CCT_CANC_TRF1 | 1 = Yes  2 = No  8 = Don't know/No answer 🡪 NA  9 = Refused 🡪 NA  -88880 = Did not complete a DCS visit 🡪 NA  -88888 = Missing 🡪 NA |
| **Type of CMC – Other CMC**  Composite variable  Has a doctor ever told you that you have had a heart attack or myocardial infarction?;  Has a doctor ever told you that you have high blood pressure or hypertension?; Has a doctor ever told you that you have dementia or Alzheimer’s disease?;. Has a doctor ever told you that you had Parkinsonism or Parkinson’s Disease?; Has a doctor ever told you that you have experienced a Stroke or CVA (cerebrovascular accident)?; Has a doctor ever told you that you have experienced a ministroke or TIA (Transient Ischemic Attack)?  CCC_AMI_COF1 and/or CCC_HBP_COF1 and/or CCC_ALZH_COF1 and/or CCC_PARK_COF1 and/or CCC_CVA_COF1 and/or CCC_TIA_COF1 and/or  CCT_AMI_TRF1 and/or CCT_HBP_TRF1 and/or CCT_ALZH_TRF1 and/or PKD_PARK_TRF1 and/or CCT_CVA_TRF1 and/or  CCT_TIA_TRF1 | 0 = No other CMC  1 = Other CMC |
| **Number of CMC**  Calculated variable  Heart disease (Y = 1, N = 0) + respiratory disorder (Y = 1, N = 0) + kidney disease (Y = 1, N = 0) + asthma (Y = 1, N = 0) + diabetes (Y = 1, N = 0) + cancer (Y = 1, N = 0) + other CMC (Y = 1, N = 0) = ∑Y = Total CMC | 0 = 0  1 = 1  2-7 = ≥2 |
| **Received professional care in past 12 months** Composite variable  During the past 12 months, did you receive short-term or long-term professional assistance at home, because of a health condition or limitation that affects your daily life, for any of the following activities?:  Received professional personal care;  Received professional medical care;  Received professional managing care;  Received professional assistance with meal preparation or delivery;  Received professional assistance with activities;  Received professional assistance with transportation;  Received professional physical therapy;  Received professional training and adaptation assistance;  Received other professional assistance  CR1_PRO_PR_COF1 and/or CR1_PRO_MD_COF1 and/or  CR1_PRO_MG_COF1 and/or CR1_PRO_MH_COF1 and/or  CR1_PRO_WK_COF1 and/or CR1_PRO_TR_COF1 and/or  CR1_PRO_PT_COF1 and/or CR1_PRO_TA_COF1 and/or  CR1_PRO_OT_COF1 and/or  CR1_PRO_PR_TRF1 and/or CR1_PRO_MD_TRF1 and/or CR1_PRO_MG_TRF1 and/or CR1_PRO_MH_TRF1 and/or CR1_PRO_WK_TRF1 and/or CR1_PRO_TR_TRF1 and/or CR1_PRO_PT_TRF1 and/or CR1_PRO_TA_TRF1 and/or CR1_PRO_OT_TRF1 | If response to any question was yes 🡪 Professional care received  If response to all questions was no 🡪 No professional care received |
| **Received non-professional care in past 12 months**  Composite variable  During the past 12 months, did you receive short-term or long-term assistance from family, friends, or neighbours because of a health condition or limitation that affects your daily life, for any of the following activities?  Received non-professional personal care;  Received non-professional medical care;  Received non-professional managing care;  Received non-professional assistance with activities;  Received non-professional assistance with transportation;  Received non-professional assistance with meal preparation;  Received non-professional physical therapy;  Received non-professional training and adaptation assistance;  Received other non-professional assistance  CR2_FAM_PR_COF1 and/or CR2_FAM_MD_COF1 and/or  CR2_FAM_MG_COF1 and/or CR2_FAM_WK_COF1 and/or  CR2_FAM_TR_COF1 and/or CR2_FAM_MH_COF1 and/or  CR2_FAM_PT_COF1 and/or CR2_FAM_TA_COF1 and/or  CR2_FAM_OT_COF1 and/or  CR2_FAM_PR_TRF1 and/or CR2_FAM_MD_TRF1 and/or  CR2_FAM_MG_TRF1 and/or CR2_FAM_WK_TRF1 and/or  CR2_FAM_TR_TRF1 and/or CR2_FAM_MH_TRF1 and/or  CR2_FAM_PT_TRF1 and/or CR2_FAM_TA_TRF1 and/or  CR2_FAM_OT_TRF1 | If response to any question was yes 🡪 Non-professional care received  If response to all questions was no 🡪 No non-professional care received |
| **Contact with family doctor**  During the past 12 months, have you had contact with any of the following about your physical or mental health?  Has had contact with: Family Doctor  HCU_FAMPHY_COF1  HCU_FAMPHY_TRF1 | 1 = Yes  2 = No  8 = Don't know/No answer 🡪 NA  9 = Refused 🡪 NA  -88888 = Missing 🡪 NA |
| **Contact with specialist**  Has had contact with: Medical specialist (such as a Cardiologist, Gynaecologist, Psychiatrist or Ophthalmologist)  HCU_SPEC_COF1  HCU_SPEC_TRF1 | 1 = Yes  2 = No  8 = Don’t know/No answer 🡪 NA  9 = Refused 🡪 NA  -88888 = Missing 🡪 NA |
| **Hospitalization**  Were you a patient in a hospital overnight during the past 12 months?  HCU_HLOVRNT_COF1  HCU_HLOVRNT_TRF1 | 1 = Yes  2 = No  8 = Don't know/No answer 🡪 NA  9 = Refused 🡪 NA  -88888 = Missing 🡪 NA |
| **Self-rated health**  In general, would you say your health is excellent, very good, good, fair, or poor?  GEN_HLTH_COF1  GEN_HLTH_TRF1 | 1 = Excellent  2 = Very good  3 = Good  4 = Fair  5 = Poor  8 = Don't know/No answer 🡪 NA  9 = Refused 🡪 NA  -88888 = Missing 🡪 NA |
| **Number in household**  How many people, not including yourself, currently live in your household?: c**alculated variable^d^**  SN_LIVH_NB_COF1  SN_LIVH_NB_TRF1 | 0 = 0  1 = 1  2-9 = ≥2  -88880 = Did not complete a DCS visit 🡪 NA  -88888 = Missing 🡪 NA |
| **Exercise**  Over the past 7 days, how often did you engage in moderate sports or recreational activities such as ballroom dancing, hunting, skating, golf without a cart, softball or other similar activities?; Over the past 7 days, how often did you engage in strenuous sports or recreational activities such as jogging, swimming, snowshoeing, cycling, aerobics, skiing or other similar activities?: **composite variable^c^**  PA2_MSPRT_COF1 and/or PA2_SSPRT_COF1 and/or  PA2_MSPRT_TRF1 and/or PA2_SSPRT_TRF1 | 1 = Never 🡪 None or Seldom  2 = Seldom (1 to 2 days) 🡪 None or Seldom  3 = Sometimes (3 to 4 days) 🡪 Sometimes or Often  4 = Often (5 to 7 days) 🡪 Sometimes or Often  8 = Don't know/No answer 🡪 NA  9 = Refused 🡪 NA  -88888 = Missing 🡪 NA |
| **Smoking status**  At the present time, do you smoke cigarettes daily, occasionally or not at all?  SMK_CURRCG_COF1  SMK_CURRCG_TRF1 | 1 = Daily (at least one cigarette every day for the past 30 days) 🡪 Daily  2 = Occasionally (at least one cigarette in the past 30 days, but not every day) 🡪 Occasionally  3 = Not at all (did not smoke at all in the past 30 days) 🡪 Not at all  8 = Don't know/No answer 🡪 NA  9 = Refused 🡪 NA  -88888 = Missing 🡪 NA |
| **Alcohol consumption**  Type of Drinker (Past 12 Months)  ALC_TTM_COF1  ALC_TTM_TRF1 | 1 = Regular drinker (at least once a month) 🡪 Regular  2 = Occasional drinker 🡪 Occasionally  3 = Did not drink in the last 12 months 🡪 Never  -77771 = Inconclusive due to at least one missing item 🡪 NA |
| **Caregiver status**  Composite variable  During the past 12 months, have you provided any of the following types of assistance to another person because of a health condition or limitation?  Provided personal care;  Provided medical care;  Provided managing care;  Provided assistance with meals or housework;  Provided assistance with house maintenance or outdoor work;  Provided assistance with transportation;  Provided social/ emotional assistance;  Provided mobility assistance;  Provided monetary assistance or financial management;  Provided other types of assistance  CAG_HLT_PR_COF1 and/or CAG_HLT_MD_COF1 and/or  CAG_HLT_MG_COF1 and/or CAG_HLT_MH_COF1 and/or CAG_HLT_WK_COF1 and/or CAG_HLT_TR_COF1 and/or  CAG_HLT_CS_COF1 and/or CAG_HLT_MB_COF1 and/or  CAG_HLT_MF_COF1 and/or CAG_HLT_OT_COF1 and/or  CAG_HLT_PR_TRF1 and/or CAG_HLT_MD_TRF1 and/or  CAG_HLT_MG_TRF1 and/or CAG_HLT_MH_TRF1 and/or  CAG_HLT_WK_TRF1 and/or CAG_HLT_TR_TRF1 and/or  CAG_HLT_CS_TRF1 and/or CAG_HLT_MB_TRF1 and/or  CAG_HLT_MF_TRF1 and/or CAG_HLT_OT_TRF1 | If response to any question was yes 🡪 Caregiver  If response to all questions was no 🡪 Non-caregiver |
| **Caregiving hours**  About how many hours per week, on average, did you spend assisting this person?  Calculated variable (reported in 1-hour increments)  CAG_HRWK_NB_COF1  CAG_HRWK_NB_TRF1 | 0-20 = 0-20  21-40 = 21-40  41+ = 41+  998 = Don't know/No answer🡪 NA  999 = Refused 🡪 NA  -88888 = Missing 🡪 NA  -99999 = Skip pattern 🡪 NA |
| **Dwelling of care recipient**  Dwelling location of person who participant provided most care giving assistance  CAG_MOST_COF1  CAG_MOST_TRF1 | 1 = Living in same household  2 = Living in another household  3 = Living in a health care institution  4 = Now deceased 🡪 NA  8 = Don't know/No answer 🡪 NA  9 = Refused 🡪 NA  -88888 = Missing 🡪 NA  -99999 = Skip pattern 🡪 NA |

*^a^COF1 indicates the Comprehensive cohort; TRF1 indicates the Tracking cohort*

**Table S2. All Models: Proportion Vaccinated and Factors Associated with Non-Vaccination Status Against Seasonal Influenza Among Participants in the First Follow-up Visit (2015-18) of the Canadian Longitudinal Study on Aging: Caregivers Aged 45 Years and Older (N=23,500)**^b^

|  | Proportion Unvaccinated among Each Response Category  (95% CI) | Model 1^a^  N=21,233 | Model 2^a^  N= 19,470 | Model 3 (Full Model)^a^  N=19,377 |
| --- | --- | --- | --- | --- |
|  |  | aOR  (95% CI) | aOR  (95% CI) | aOR  (95% CI) |
| Age (Years) |  |  |  |  |
| 85-92 | 0.18 (0.15, 0.21) | Ref | Ref | Ref |
| 75-84 | 0.22 (0.21, 0.24) | 1.32 (1.05, 1.66) | 1.46 (1.14, 1.87) | 1.42 (1.11, 1.82) |
| 65-74 | 0.34 (0.33, 0.35) | 2.39 (1.91, 2.99) | 2.58 (2.03, 3.29) | 2.43 (1.91, 3.09) |
| 55-64 | 0.50 (0.49, 0.51) | 4.90 (3.91, 6.14) | 5.21 (4.09, 6.65) | 4.71 (3.70, 6.00) |
| 46-54 | 0.59 (0.58, 0.61) | 6.67 (5.27, 8.44) | 7.11 (5.52, 9.16) | 6.29 (4.89, 8.11) |
| Province of Residence |  |  |  |  |
| Ontario | 0.37 (0.36, 0.39) | Ref | Ref | Ref |
| Newfoundland | 0.45 (0.42, 0.47) | 1.35 (1.18, 1.56) | 1.35 (1.17, 1.56) | 1.41 (1.22, 1.63) |
| Prince Edward Island | 0.34 (0.30, 0.39) | 0.78 (0.62, 0.99) | 0.77 (0.60, 0.99) | 0.75 (0.59, 0.97) |
| Nova Scotia | 0.29 (0.27, 0.31) | 0.58 (0.51, 0.66) | 0.58 (0.50, 0.66) | 0.58 (0.51, 0.67) |
| New Brunswick | 0.37 (0.33, 0.41) | 0.84 (0.68, 1.05) | 0.85 (0.67, 1.06) | 0.83 (0.66, 1.05) |
| Quebec | 0.55 (0.54, 0.57) | 1.99 (1.82, 2.18) | 1.95 (1.77, 2.15) | 1.94 (1.76, 2.14) |
| Manitoba | 0.42 (0.40, 0.44) | 1.10 (0.98, 1.24) | 1.09 (0.97, 1.23) | 1.09 (0.97, 1.24) |
| Saskatchewan | 0.40 (0.36, 0.44) | 1.05 (0.86, 1.27) | 1.01 (0.82, 1.25) | 1.00 (0.81, 1.23) |
| Alberta | 0.37 (0.35, 0.39) | 0.96 (0.86, 1.07) | 0.95 (0.85, 1.06) | 0.95 (0.85, 1.07) |
| British Columbia | 0.41 (0.4, 0.43) | 1.18 (1.08, 1.29) | 1.19 (1.08, 1.30) | 1.21 (1.10, 1.33) |
| Sex |  |  |  |  |
| Male | 0.41 (0.40, 0.42) | Ref | Ref | Ref |
| Female | 0.42 (0.41, 0.42) | 0.87 (0.82, 0.92) | 1.15 (1.08, 1.23) | 1.14 (1.07, 1.22) |
| Urban or Rural |  |  |  |  |
| Rural | 0.47 (0.45, 0.48) | Ref | Ref | Ref |
| Urban | 0.41 (0.40, 0.41) | 1.32 (1.21, 1.43) | 0.75 (0.69, 0.82) | 0.76 (0.70, 0.83) |
| Household Income (Canadian Dollars) |  |  |  |  |
| <20000 | 0.48 (0.45, 0.51) | Ref | Ref | Ref |
| ≥20000 to <50000 | 0.42 (0.40, 0.43) | 0.85 (0.72, 1.00) | 0.84 (0.71, 1.00) | 0.87 (0.73, 1.04) |
| ≥50000 to <100000 | 0.39 (0.38, 0.40) | 0.60 (0.51, 0.71) | 0.59 (0.50, 0.70) | 0.63 (0.53, 0.75) |
| ≥100000 to <150000 | 0.43 (0.41, 0.44) | 0.55 (0.46, 0.65) | 0.53 (0.44, 0.63) | 0.57 (0.47, 0.69) |
| ≥150000 | 0.44 (0.42, 0.45) | 0.47 (0.40, 0.56) | 0.45 (0.38, 0.55) | 0.50 (0.41, 0.60) |
| Education |  |  |  |  |
| Less than secondary school graduation | 0.40 (0.37, 0.43) | Ref | Ref | Ref |
| Secondary school graduation, no post-secondary education | 0.44 (0.42, 0.46) | 1.02 (0.86, 1.21) | 1.08 (0.90, 1.29) | 1.09 (0.91, 1.31) |
| Some post-secondary education | 0.43 (0.40, 0.45) | 1.05 (0.88, 1.26) | 1.09 (0.90, 1.32) | 1.11 (0.92, 1.34) |
| Post-secondary degree/diploma | 0.41 (0.40, 0.42) | 0.85 (0.73, 0.98) | 0.89 (0.76, 1.05) | 0.93 (0.79, 1.09) |
| Race |  |  |  |  |
| White | 0.41 (0.40, 0.42) | Ref | Ref | Ref |
| Non-white | 0.50 (0.47, 0.53) | 1.41 (1.22, 1.63) | 1.42 (1.22, 1.65) | 1.42 (1.22, 1.65) |
| CMC by Type |  |  |  |  |
| Heart Disease | 0.29 (0.27, 0.30) | 0.80 (0.72, 0.89) | 0.79 (0.71, 0.89) | 0.90 (0.79, 1.02) |
| Respiratory disorders | 0.31 (0.29, 0.34) | 0.80 (0.70, 0.91) | 0.79 (0.69, 0.90) | 0.85 (0.73, 0.99) |
| Kidney Disease or Failure | 0.33 (0.30, 0.37) | 0.87 (0.73, 1.04) | 0.86 (0.71, 1.04) | 0.95 (0.78, 1.15) |
| Asthma | 0.34 (0.33, 0.36) | 0.65 (0.60, 0.71) | 0.66 (0.60, 0.72) | 0.73 (0.65, 0.83) |
| Diabetes | 0.33 (0.31, 0.34) | 0.71 (0.66, 0.77) | 0.71 (0.66, 0.78) | 0.79 (0.70, 0.89) |
| Cancer | 0.31 (0.29, 0.32) | 0.77 (0.71, 0.84) | 0.79 (0.72, 0.85) | 0.89 (0.79, 1.00) |
| Other CMC | 0.34 (0.33, 0.35) | 0.77 (0.72, 0.82) | 0.78 (0.73, 0.84) | 0.88 (0.78, 0.99) |
| Number in Household Besides Participant |  |  |  |  |
| 0 | 0.39 (0.37, 0.40) | Ref | Ref | Ref |
| 1 | 0.38 (0.38, 0.39) | 0.98 (0.90, 1.06) | 1.01 (0.93, 1.10) | 1.01 (0.93, 1.11) |
| ≥ 2 | 0.51 (0.49, 0.52) | 1.16 (1.05, 1.28) | 1.22 (1.10, 1.36) | 1.21 (1.09, 1.35) |
| Hours of Care Provided Weekly |  |  |  |  |
| 1-20 | 0.42 (0.41, 0.43) |  | Ref | Ref |
| 21-40 | 0.39 (0.37, 0.41) |  | 0.92 (0.81, 1.04) | 0.93 (0.82, 1.05) |
| 41+ | 0.39 (0.36, 0.41) |  | 1.01 (0.89, 1.16) | 1.01 (0.88, 1.15) |
| Location of Care Recipients |  |  |  |  |
| Living in Your Household | 0.36 (0.35, 0.37) |  | Ref | Ref |
| Living in Another Household | 0.43 (0.43, 0.44) |  | 1.17 (1.08, 1.27) | 1.16 (1.07, 1.26) |
| Living in a Health Care Institution | 0.41 (0.39, 0.43) |  | 1.13 (1.01, 1.27) | 1.13 (1.00, 1.27) |
| Number of CMC |  |  |  |  |
| 0 | 0.51 (0.50, 0.53) |  |  | Ref |
| 1 | 0.42 (0.41, 0.43) |  |  | 0.97 (0.86, 1.09) |
| ≥ 2 | 0.30 (0.29, 0.31) |  |  | 0.83 (0.66, 1.03) |
| Type of Healthcare Utilization Past 12 months |  |  |  |  |
| Family Doctor Contact | 0.39 (0.38, 0.40) |  |  | 0.53 (0.47, 0.59) |
| Specialist Contact | 0.36 (0.35, 0.37) |  |  | 0.81 (0.76, 0.86) |
| Self-Rated Health |  |  |  |  |
| Excellent | 0.45 (0.43, 0.46) |  |  | Ref |
| Very Good | 0.42 (0.41, 0.43) |  |  | 0.99 (0.91, 1.08) |
| Good | 0.41 (0.40, 0.42) |  |  | 0.96 (0.87, 1.06) |
| Fair | 0.36 (0.34, 0.38) |  |  | 0.88 (0.76, 1.01) |
| Poor | 0.32 (0.28, 0.37) |  |  | 0.78 (0.59, 1.03) |
| Exercise Past Week |  |  |  |  |
| None or Seldom | 0.41 (0.40, 0.41) |  |  | Ref |
| Sometimes or Often | 0.44 (0.42, 0.45) |  |  | 1.00 (0.93, 1.08) |
| Smoking Currently |  |  |  |  |
| Not at All | 0.40 (0.40, 0.41) |  |  | Ref |
| Occasionally | 0.52 (0.47, 0.57) |  |  | 1.01 (0.79, 1.29) |
| Daily | 0.56 (0.53, 0.58) |  |  | 1.44 (1.25, 1.65) |
| Alcohol Past 12 Months |  |  |  |  |
| Never | 0.41 (0.39, 0.43) |  |  | Ref |
| Occasionally | 0.42 (0.41, 0.44) |  |  | 1.07 (0.94, 1.22) |
| Regular | 0.41 (0.41, 0.42) |  |  | 0.92 (0.84, 1.02) |

^a^Grey cells indicate variables that were not included in the model represented by that column.

^b^Model 1 of the nested models included sociodemographic factors, CMC categories, and the transmission-associated variable of household size. Model 2 included variables about the caregivers’ relationship to their care recipient(s) as well as the Model 1 covariates. Model 3, the full model, included health-related variables (number of CMC, healthcare utilization, self-rated health, and health behaviors) along with the covariates in Model 2.

**Table S3. All Models: Proportion Vaccinated and Factors Associated with Non-Vaccination Status Against Seasonal Influenza Among Participants in the First Follow-up Visit (2015-18) of the Canadian Longitudinal Study on Aging: Care Recipients Aged 65 Years and Older (N=5,559)**^b^

|  | Proportion Unvaccinated among Each Response Category  (95% CI) | Model 1^a^  N=4,570 | Model 2^a^  N=4,552 | Model 3 (Full Model)^a^  N=4,521 |
| --- | --- | --- | --- | --- |
|  |  | aOR  (95% CI) | aOR  (95% CI) | aOR  (95% CI) |
| Age (Years) |  |  |  |  |
| 85-92 | 0.21 (0.18, 0.23) | Ref | Ref | Ref |
| 75-84 | 0.21 (0.19, 0.22) | 1.07 (1.58, 2.53) | 1.07 (0.84, 1.36) | 1.05 (0.83, 1.34) |
| 65-74 | 0.31 (0.29, 0.32) | 2.00 (0.84, 1.36) | 1.96 (1.55, 2.49) | 1.88 (1.48, 2.39) |
| Province of Residence |  |  |  |  |
| Ontario | 0.20 (0.17, 0.22) | Ref | Ref | Ref |
| Newfoundland | 0.32 (0.26, 0.38) | 1.98 (1.41, 2.78) | 1.94 (1.38, 2.74) | 1.99 (1.41, 2.82) |
| Prince Edward Island | 0.19 (0.12, 0.25) | 0.81 (0.47, 1.39) | 0.82 (0.48, 1.40) | 0.84 (0.49, 1.43) |
| Nova Scotia | 0.15 (0.12, 0.19) | 0.70 (0.50, 1.00) | 0.70 (0.50, 0.99) | 0.71 (0.50, 1.01) |
| New Brunswick | 0.27 (0.20, 0.35) | 1.19 (0.74, 1.90) | 1.15 (0.72, 1.84) | 1.13 (0.70, 1.81) |
| Quebec | 0.33 (0.31, 0.36) | 1.84 (1.48, 2.30) | 1.83 (1.46, 2.28) | 1.90 (1.52, 2.38) |
| Manitoba | 0.26 (0.23, 0.30) | 1.14 (0.84, 1.54) | 1.14 (0.84, 1.54) | 1.16 (0.86, 1.58) |
| Saskatchewan | 0.28 (0.21, 0.36) | 1.48 (0.92, 2.37) | 1.44 (0.89, 2.31) | 1.45 (0.90, 2.33) |
| Alberta | 0.20 (0.17, 0.23) | 0.96 (0.73, 1.27) | 0.96 (0.72, 1.27) | 0.98 (0.74, 1.30) |
| British Columbia | 0.27 (0.24, 0.29) | 1.45 (1.17, 1.81) | 1.44 (1.15, 1.79) | 1.45 (1.16, 1.81) |
| Sex |  |  |  |  |
| Male | 0.24 (0.22, 0.25) | Ref | Ref | Ref |
| Female | 0.26 (0.24, 0.27) | 1.07 (0.92, 1.24) | 1.06 (0.91, 1.23) | 1.09 (0.93, 1.27) |
| Urban or Rural |  |  |  |  |
| Rural | 0.29 (0.26, 0.32) | Ref | Ref | Ref |
| Urban | 0.24 (0.23, 0.25) | 0.76 (0.62, 0.93) | 0.77 (0.63, 0.95) | 0.77 (0.62, 0.94) |
| Household Income (Canadian Dollars) |  |  |  |  |
| < 20000 | 0.37 (0.33, 0.41) | Ref | Ref | Ref |
| ≥20000 to <50000 | 0.29 (0.27, 0.31) | 0.73 (0.58, 0.92) | 0.75 (0.59, 0.95) | 0.78 (0.61, 0.99) |
| ≥50000 to <100000 | 0.21 (0.19, 0.23) | 0.47 (0.37, 0.60) | 0.49 (0.38, 0.63) | 0.52 (0.40, 0.68) |
| ≥100000 to <150000 | 0.17 (0.13, 0.20) | 0.34 (0.24, 0.47) | 0.36 (0.26, 0.50) | 0.38 (0.27, 0.55) |
| ≥150000 | 0.17 (0.13, 0.22) | 0.36 (0.24, 0.53) | 0.38 (0.26, 0.57) | 0.42 (0.27, 0.64) |
| Education |  |  |  |  |
| Less than secondary school graduation | 0.29 (0.25, 0.33) | Ref | Ref | Ref |
| Secondary school graduation, no post-secondary education | 0.26 (0.23, 0.29) | 0.90 (0.66, 1.22) | 0.91 (0.67, 1.24) | 0.93 (0.68, 1.26) |
| Some post-secondary education | 0.27 (0.23, 0.31) | 1.20 (0.87, 1.66) | 1.26 (0.91, 1.74) | 1.28 (0.92, 1.78) |
| Post-secondary degree/diploma | 0.24 (0.22, 0.25) | 0.89 (0.69, 1.14) | 0.93 (0.72, 1.20) | 0.94 (0.73, 1.22) |
| Race |  |  |  |  |
| white | 0.24 (0.23, 0.26) | Ref | Ref | Ref |
| Non-white | 0.36 (0.29, 0.43) | 2.19 (1.51, 3.18) | 2.19 (1.50, 3.19) | 2.04 (1.39, 2.99) |
| CMC by Type |  |  |  |  |
| Heart Disease | 0.22 (0.20, 0.25) | 0.81 (0.68, 0.96) | 0.87 (0.71, 1.06) | 0.87 (0.72, 1.07) |
| Respiratory Disorders | 0.20 (0.17, 0.23) | 0.62 (0.49, 0.78) | 0.66 (0.52, 0.83) | 0.63 (0.49, 0.81) |
| Kidney Disease or Failure | 0.21 (0.17, 0.25) | 0.75 (0.56, 1.01) | 0.78 (0.58, 1.05) | 0.78 (0.57, 1.05) |
| Asthma | 0.23 (0.20, 0.26) | 0.92 (0.75, 1.13) | 0.94 (0.75, 1.18) | 0.97 (0.77, 1.22) |
| Diabetes | 0.23 (0.21, 0.25) | 0.82 (0.69, 0.96) | 0.87 (0.72, 1.05) | 0.87 (0.72, 1.06) |
| Cancer | 0.22 (0.20, 0.24) | 0.84 (0.72, 0.98) | 0.89 (0.74, 1.08) | 0.88 (0.72, 1.06) |
| Other CMC | 0.24 (0.22, 0.25) | 0.85 (0.73, 0.99) | 0.89 (0.72, 1.10) | 0.88 (0.71, 1.09) |
| Number of CMC |  |  |  |  |
| 0 | 0.29 (0.26, 0.32) |  | Ref | Ref |
| 1 | 0.28 (0.25, 0.30) |  | 1.11 (0.85, 1.44) | 1.10 (0.85, 1.44) |
| ≥ 2 | 0.22 (0.21, 0.24) |  | 0.93 (0.63, 1.38) | 0.92 (0.62, 1.36) |
| Care or Assistance Received |  |  |  |  |
| Professional | 0.23 (0.21, 0.24) |  | 0.91 (0.76, 1.09) | 0.89 (0.74, 1.06) |
| Non-Professional | 0.25 (0.24, 0.27) |  | 1.10 (0.89, 1.36) | 1.08 (0.87, 1.34) |
| Type of Healthcare Utilization Past 12 Months |  |  |  |  |
| Family Doctor Contact | 0.24 (0.23, 0.25) |  | 0.61 (0.41, 0.89) | 0.61 (0.41, 0.90) |
| Specialist Contact | 0.23 (0.22, 0.24) |  | 0.70 (0.58, 0.85) | 0.71 (0.59, 0.86) |
| Hospitalization History | 0.25 (0.23, 0.27) |  | 1.15 (0.98, 1.35) | 1.16 (0.98, 1.36) |
| Self-Rated Health |  |  |  |  |
| Excellent | 0.28 (0.24, 0.32) |  |  | Ref |
| Very Good | 0.25 (0.23, 0.27) |  |  | 0.99 (0.75, 1.29) |
| Good | 0.24 (0.22, 0.26) |  |  | 0.94 (0.72, 1.23) |
| Fair | 0.25 (0.23, 0.28) |  |  | 1.10 (0.81, 1.48) |
| Poor | 0.21 (0.17, 0.26) |  |  | 0.84 (0.56, 1.25) |
| Number in Household Besides Participant |  |  |  |  |
| 0 | 0.27 (0.25, 0.29) |  |  | Ref |
| 1 | 0.23 (0.21, 0.25) |  |  | 0.99 (0.83, 1.17) |
| ≥ 2 | 0.26 (0.22, 0.30) |  |  | 1.11 (0.85, 1.45) |
| Exercise Past Week |  |  |  |  |
| None or Seldom | 0.24 (0.23, 0.26) |  |  | Ref |
| Sometimes or Often | 0.27 (0.24, 0.31) |  |  | 1.08 (0.88, 1.34) |
| Smoking Currently |  |  |  |  |
| Not at All | 0.24 (0.23, 0.25) |  |  | Ref |
| Occasionally | 0.33 (0.19, 0.48) |  |  | 1.43 (0.66, 3.12) |
| Daily | 0.34 (0.29, 0.40) |  |  | 1.46 (1.06, 1.99) |
| Alcohol Past 12 Months |  |  |  |  |
| Never | 0.27 (0.25, 0.30) |  |  | Ref |
| Occasionally | 0.28 (0.25, 0.31) |  |  | 1.07 (0.85, 1.35) |
| Regular | 0.23 (0.22, 0.25) |  |  | 0.90 (0.74, 1.09) |

^a^Grey cells indicate variables that were not included in the model represented by that column.

^b^Our nested models first estimated the associations between influenza non-vaccination and sociodemographic variables and self-reported categories of CMC in Model 1. In Model 2, we added the objective healthcare-related variables of *number* of CMC (0, 1, or ≥2), type of care received (professional or non), and healthcare utilization variables to the covariates included in Model 1. Finally, in Model 3, the full model, we added variables also associated with health and influenza transmission— self-rated health, household size, and health behaviors— to the covariates included in Model 2.
